# Percutaneous insertion of peritoneal dialysis catheter is a safe and effective technique irrespective of BMI

**DOI:** 10.1186/s12882-020-01850-5

**Published:** 2020-05-25

**Authors:** Dayang Xie, Jianhui Zhou, Xueying Cao, Qingtao Zhang, Yanli Sun, Li Tang, Jing Huang, Juanli Zheng, Li Lin, Zhenzhen Li, Guangyan Cai, Xiangmei Chen

**Affiliations:** Department of Nephrology, the First Medical Centre, Chinese PLA General Hospital, Chinese PLA Institute of Nephrology, State Key Laboratory of Kidney Diseases (2011DAV00088), National Clinical Research Center for Kidney Diseases, Fuxing Road 28, Beijing, 100853 People’s Republic of China

**Keywords:** Peritoneal dialysis catheter insertion, Percutaneous, Complication, Survival, BMI

## Abstract

**Background:**

A large body mass index (BMI) has been considered as a relative contraindication for percutaneous catheter insertion, although this technique has many advantages. Up to now, there are few studies on peritoneal catheter placement and obesity. The aim of this study was to determine whether patients with large BMI can also choose the percutaneous technique for peritoneal dialysis catheter insertion.

**Methods:**

One hundred eighty seven consecutive patients underwent peritoneal catheter insertions in the Chinese PLA General Hospital between January 1, 2015 and December 31, 2016, with 178 eligible cases being included in the analysis. Two groups were created based on the catheter insertion techniques, the percutaneous group (group P) and the surgical group (group S). Subgroups were created according to BMI > 28 or ≤ 28. The outcomes included catheter related complications and catheter survival.

**Results:**

Total infectious complication rates were significantly lower in group P than in group S. There were no significant differences in peritonitis rate between group P and group S (1.20% vs. 3.16% with *P* = 0.71 in early stage, and 4.82% vs. 11.58% with *P* = 0.11 in late stage). All other measured complications were similar between the two groups. Though the one-year infection-free catheter survival in group P was 7.5% higher than group S, the difference was not significant. The one-year dysfunction-free catheter survival, one-year dysfunction-and-infection-free catheter survival, and overall catheter survival were similar between the two groups. Subgroup analyses showed a superior one-year infection-free catheter survival of percutaneous technique in patients with BMI > 28, which was confirmed by Kaplan-Meier analysis.

**Conclusions:**

Despite the challenges that may be encountered with patients who have a large BMI, the percutaneous technique seems to be a safe and effective approach to placing a peritoneal dialysis catheter.

## Background

Peritoneal dialysis (PD) is one of the major renal replacement therapies for end stage renal disease, which compared to hemodialysis has the added benefits of preserving residual renal function, offering a better quality of life, less expensive, minimal infrastructure required, hemodynamic stability and similar survival [1–5]. Peritoneal dialysis is a preferred modality for patients expecting to receive a transplant [6], it can be used for urgent-start dialysis [7], and possible earlier recovery of kidney function in acute kidney injury [8].

One of the keys to successful PD and avoidance of urgent hemodialysis is creating access to timely insertion of a well-functioning peritoneal dialysis catheter [9–11]. Currently, there are several techniques available for PD catheter placement which include surgical, laparoscopic and percutaneous [10].. Each technique has its own advantages and shortcomings. Compared with other methods, percutaneous catheter insertion is a simple and shorter procedure with low complication rate. It requires minimal training to perform and can offer early recovery from the procedure. Thus, percutaneous insertion of PD catheters should theoretically be a preferred choice of technique by primary nephrologists. However, a large BMI has been considered a relative contraindication to percutaneous insertion [12–16], which has limited its use in obese patients. Currently, the number of people suffering from overweight and obesity continues to rise, but there are few studies on peritoneal catheter placement techniques and obesity. The aim of this study was to compare the outcomes of percutaneous and surgically placed PD catheters on patients with a large BMI (> 28), with the primary outcome being the overall catheter survival and secondary outcomes being non-infectious complication rates, infectious complication rates, catheter dysfunction-free survival, catheter infection-free survival, catheter dysfunction-and-infection-free survival.

## Methods

### Study design

**Fig. 1:**
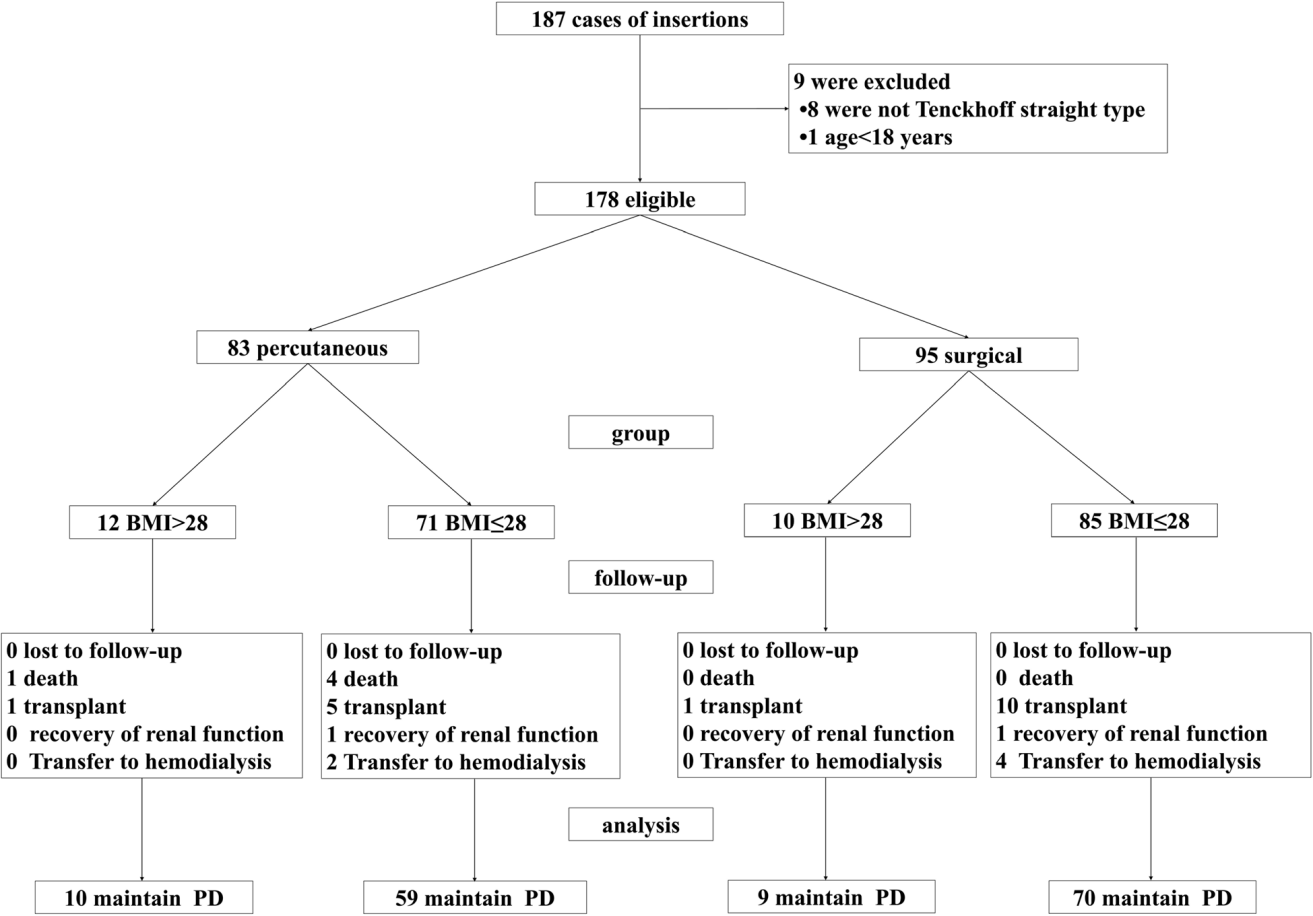
Flow chart of participants. Totally 187 consecutive patients who underwent PD catheter insertions were examined. Then 178 eligible patients were grouped by the insertion technique. Subgroups were created by BMI. Then the patients were followed up for one year and the outcomes (complications and catheter survivals) were compared. BMI, body mass index

We retrospectively examined collected data on 187 consecutive patients who had undergone surgical technique and percutaneous technique peritoneal catheter insertions in the Chinese PLA General Hospital between January 1, 2015 and December 31, 2016. Inclusion criteria: 1. age ≥ 18 years; 2. the catheter style was a straight Tenckhoff. Exclusion criteria: the medical record was incomplete, such as lack of BMI value, complications or survival. A total of 178 eligible cases were included in the analysis. Two groups were created based on the catheter insertion techniques: percutaneous group (group P, *n* = 83) and surgical group (group S, *n* = 95). Subgroups were created according to BMI. Subgroup A consisted of 22 obese patients with BMI > 28, 12 percutaneous and 10 surgical. Subgroup B consisted of 156 patients with a BMI ≤ 28, 71 percutaneous and 85 surgical (Fig. 1). The outcomes of percutaneous and surgical techniques were compared and the effect of BMI was determined.

### Patient characteristics and data sources

All patients had a clear clinical diagnosis of underlying renal disease and required renal replacement therapy prior to a PD catheter insertion. The catheters were placed percutaneously or surgically by three experienced nephrologists from the PD center of Chinese PLA General Hospital who used the same operation approach. Catheter insertions were performed according to the published protocols [17–19]. Percutaneous insertions were performed at the bedside within 24 h to 48 h after admission. Surgical insertions were performed in a sterile operating room, on average, five to 7 days after admission due to limited operating room availability. The choice of catheter insertion method was not randomized but rather decided by the preference of the physician and patient. The percutaneous technique was commonly chosen for patients who were elderly, immobile, medically unstable or requiring urgent dialysis. All percutaneous and most surgical insertions were performed under local anesthesia. Prophylactic antibiotics (first or second generation cephalosporin) and phenobarbital sodium intramuscular were routinely given prior to the procedure. The data source was the medical records of the regularly scheduled follow-up registration system of our PD program. Patient follow-up and medical records were written by our PD doctors and nurses.

### Outcomes and definitions

The measured outcomes included: non-infectious complication rates, infectious complication rates, catheter dysfunction-free survival, catheter infection-free survival, catheter dysfunction-and-infection-free survival, and overall catheter survival. Patient survival was not included as an outcome as the 5 deaths were not related to catheter insertion technique. Instead, patient death was a censoring condition of catheter survival. We defined complications occurring within 30 days (non-infectious) or within 2 weeks (infectious) after catheter insertion as early complications, otherwise described as late [19, 20]. The non-infectious complications consisted of mechanical catheter dysfunction, leakage, hernia development, bleeding, visceral injury and insertion failure. Mechanical catheter dysfunction was defined as inflow/outflow obstruction, catheter tip migration, omental wrap, and blood or fibrin clot requiring revisionary surgery or catheter removal. The infectious complications consisted of peritonitis, catheter related infections (exit-site and tunnel infection). The definition of peritonitis, exit-site and tunnel infection complied with the ISPD guideline [21]. Catheter dysfunction-free survival was defined as total length of time from catheter insertion to revisionary surgery or removal due to mechanical dysfunction only. The case was censored if lost to follow-up, death, catheter removal for other reasons, i.e., peritonitis, catheter related infections, ultrafiltration failure, renal transplant, renal recovery, or patient’s preference for hemodialysis. Catheter infection-free survival was defined as total length of time from catheter insertion to peritonitis or catheter related infections. Catheter dysfunction-and-infection-free survival was defined as total length of time from catheter insertion either to surgery revision or removal due to mechanical dysfunction, or infectious complications. The overall catheter survival was defined as total length of time from catheter insertion to removal. The cases lost to follow-up, death, renal transplanted, or renal recovery were censored. Each patient was followed up for 1 year from the day of catheter insertion.

### Statistical analysis

SPSS 17.0 was used for statistical analysis, continuous variables were represented by mean ± standard deviation, non-continuous variables were expressed as percentage. Comparisons between groups were conducted by independent t-test or chi-square test, the subgroup analysis was conducted by stratified chi-square test, and catheter survival was assessed using the Kaplan–Meier method with log-rank test. Statistical significance was established at 0.05.

## Results

### Characteristics of participants

The cohort comprised 187 consecutive PD catheter insertions from January 1, 2015 to December 31, 2016, while 178 eligible cases were included in the analysis. The baseline characteristics are shown in Table 1. The mean age of group P was significantly higher than that of group S (48.07 ± 16.84 years vs. 43.22 ± 13.13 years, *P* = 0.04). There were no significant differences in mean weight or mean BMI. Sex ratio of group P was similar to that of group S, 62.65% (52 cases) and 66.32% (63 cases) of male, respectively, *P* = 0.61. There was a higher incidence of carotid atherosclerosis in group P than in group S, 72.29% (60 cases) vs. 37.89% (36 cases), *P* < 0.001. Respiratory disease was more commonly observed in group P (13.25%, 11 cases) than in group S (none), *P* < 0.001. There was no significant difference in other comorbidities, such as diabetes, hypertension, coronary heart disease or cerebrovascular disease.

**Table 1:** Baseline patient characteristics at PD catheter insertion

Characteristics	Percutaneous (*n* = 83)	Surgical (*n* = 95)	*P*-value
Sex [n (%)]			
Male	52 (62.65)	63 (66.32)	0.61
Female	31 (37.35)	32 (33.68)	
Age (years)	48.07 ± 16.84	43.22 ± 13.13	0.04
≥ 65 years [n (%)]	28 (33.73)	4 (4.21)	< 0.001
Weight (kg)	65.97 ± 15.34	66.87 ± 13.34	0.67
BMI (kg/m2)	23.44 ± 4.02	23.48 ± 3.84	0.95
> 28 [n (%)]	12 (14.46)	10 (10.53)	0.43
≤ 28 [n (%)]	71 (85.54)	85 (89.47)	
Serum albumin (g/L)	36.20 ± 4.40	37.76 ± 4.24	0.02
Serum creatinine (μmol/L)	886.66 ± 324.41	791.37 ± 230.15	0.03
eGFR (CKD-EPI) [mL/(min·1.73 m2)]	5.69 ± 2.08	6.47 ± 2.01	0.01
Acute kidney injury [n (%)]	1 (1.20)	1 (1.05)	
Chronic kidney diseases [n (%)]	82 (98.80)	94 (98.95)	0.92
Comorbidity [n (%)]			
Hypertension	79 (95.18)	83 (87.37)	0.07
Diabetes	13 (15.66)	13 (13.68)	0.71
Coronary artery disease	9 (10.84)	5 (5.26)	0.17
Cerebrovascular disease	7 (8.43)	6 (6.32)	0.59
Carotid atherosclerosis	60 (72.29)	36 (37.89)	< 0.001
Respiratory disease	11 (13.25)	0	< 0.001
Chronic bronchitis	4 (4.82)	0	
Old pulmonary tuberculosis	1 (1.20)	0	
Bronchial asthma	3 (3.61)	0	
Bronchiectasis	1 (1.20)	0	
Interstitial lung disease	1 (1.20)	0	
Sleep apnea hypopnea syndrome	1 (1.20)	0	
Planned catheter insertion [n (%)]	60 (72.29)	95 (100)	< 0.001

Continuous variables are presented as mean ± SD, while non-continuous variables are presented as number (percentage). Weight was examined under standard conditions, i.e., after urinating and defecating with empty peritoneal cavity. Hypertension was judged by 140/90 mmHg or 150/90 mmHg (age ≥ 60). Carotid atherosclerosis was determined by ultrasound. *BMI* Body mass index

### Non-infectious complications

Non-infectious complications of the two groups are shown in Table 2. In the present study, none of the patients had insertion failure, visceral injury, leakage, severe bleeding, or hernia development during the early period. Late stage hernia development was observed more commonly in group S (4.21%, 4 cases) than in group P (none), though of no statistical significance. Early mechanical catheter dysfunction rates were similar between the two groups (9.64 and 9.47% for group P and S respectively, *P* = 0.97). Late mechanical dysfunction rates were as well similar (2.41 and 3.16% for group P and S respectively, *P* = 1.00). Total non-infectious complication rates were similar between the two groups (12.05 and 16.84% for group P and S respectively, *P* = 0.37). The results of subgroup analysis (shown in Table 3) were similar to the above.

**Table 2:** Complications over one year of follow-up

Complications	Percutaneous (*n* = 83)	Surgical (*n* = 95)	*P*-value
Non-infectious complications			
Early [n (%)]			
Mechanical catheter dysfunction	8 (9.64)	9 (9.47)	0.97
Dialysate leakage	0	0	–
Hernia	0	0	–
Bleeding	0	0	–
Visceral injury	0	0	–
Insertion failure	0	0	–
Late [n (%)]			
Mechanical catheter dysfunction	2 (2.41)	3 (3.16)	1.00
Dialysate leakage	0	0	–
Hernia	0	4 (4.21)	0.17
Bleeding	0	0	–
Total [n (%)]	10 (12.05)	16 (16.84)	0.37
Infectious complications			
Early [n (%)]			
Peritonitis	1 (1.20)	3 (3.16)	0.71
Catheter related infections	0	0	–
Late [n (%)]			
Peritonitis	4 (4.82)	11 (11.58)	0.11
Catheter related infections	0	1 (1.05)	1.00
Total [n (%)]	5 (6.02)	15 (15.79)	0.04

Variables are presented as number and percentage (if number is not 0). The upper half of the table is non-infectious complications, and the lower half is infectious complications. Both non-infectious and infectious complications are divided into three parts, i.e., early stage, late stage and total. Early was defined as complications occurred within 30 days after catheter insertion (for non-infectious), or 2 weeks after insertion (for infectious). Bleeding means only severe conditions when demanding transfusion or surgical hemostasis. Catheter related infection consists of exit site and tunnel infections

**Table 3:** Subgroup analysis for the complications by BMI (Percutaneous versus Surgical)

Complications	Percutaneous (*n* = 83)		Surgical (*n* = 95)		OR_MH_ (95% CI)	*P*-value
	BMI > 28 (*n* = 12)	BMI ≤ 28 (*n* = 71)	BMI > 28 (*n* = 10)	BMI ≤ 28 (*n* = 85)		
Non-infectious complications						
Early [n (%)]						
Mechanical PD catheter dysfunction	0	8 (5.13)	1 (4.55)	8 (5.13)	1.05 (0.39 to 2.83)	0.93
Dialysate leak	0	0	0	0	–	–
Hernia	0	0	0	0	–	–
Bleeding	0	0	0	0	–	–
Visceral injury	0	0	0	0	–	–
Insertion failure	0	0	0	0	–	–
Late [n (%)]						
Mechanical PD catheter dysfunction	1 (4.55)	1 (0.64)	0	3 (1.92)	0.73 (0.12 to 4.62)	0.74
Dialysate leak	0	0	0	0	–	–
Hernia	0	0	0	4 (2.56)	0.00	0.06
Bleeding	0	0	0	0	–	–
Total [n (%)]	1 (4.55)	9 (5.77)	1 (4.55)	15 (9.62)	0.69 (0.29 to 1.62)	0.39
Infectious complications						
Early (n)						
Peritonitis	0	1 (0.64)	1 (4.55)	2 (1.28)	0.37 (0.04 to 3.45)	0.35
Catheter related infections	0	0	0	0	–	–
Late [n (%)]						
Peritonitis	1 (4.55)	3 (1.92)	3 (13.64)	8 (5.13)	0.36 (0.11 to 1.19)	0.08
Catheter related infections	0	0	1 (4.55)	0	0.00	0.26
Total (n)	1 (4.55)	4 (2.56)	5 (22.73)	10 (6.41)	0.32 (0.11 to 0.91)	0.02

Variables are presented as number and percentage (if number is not 0). The percentage is the proportion of complications in the relevant subgroup. Subgroup analysis was performed by stratified chi-square test. The upper half of the table is non-infectious complications, and the lower half is infectious complications. Both non-infectious and infectious complications are divided into three parts, i.e., early stage, late stage and total. Early was defined as complications occurred within 30 days after catheter insertion (for non-infectious), or 2 weeks after insertion (for infectious). Bleeding means only severe conditions when demanding transfusion or surgical hemostasis. Catheter related infection consists of exit site and tunnel infections. Subgroup A: data in the two columns of BMI > 28, subgroup B: data in the two columns of BMI ≤ 28. *BMI* Body mass index

### Infectious complications

Infectious complications of the two groups are shown in Table 2. Early stage catheter related infections were not observed in group P or S. Late stage catheter related infections were similar between group P (none) and group S (1.05%), *P* = 1.00. There were no significant differences in peritonitis rate between the two groups (1.20% vs. 3.16% with *P* = 0.71 in early stage, and 4.82% vs. 11.58% with *P* = 0.11 in late stage). Total infectious complication rate was lower in group P (6.02%) than in group S (15.79%), *P* = 0.04.

The results of subgroup analysis are shown in Table 3. Between different subgroups, only the total infectious complication distribution was significantly different (*P* = 0.02). This difference was driven mainly by subgroup A in which the total infectious complication rate of group P (4.55%) was lower than that of group S (22.73%). Both the early and the late infectious complication distributions showed no difference between subgroup A and B.

### One-year catheter survival

**Fig. 2:**
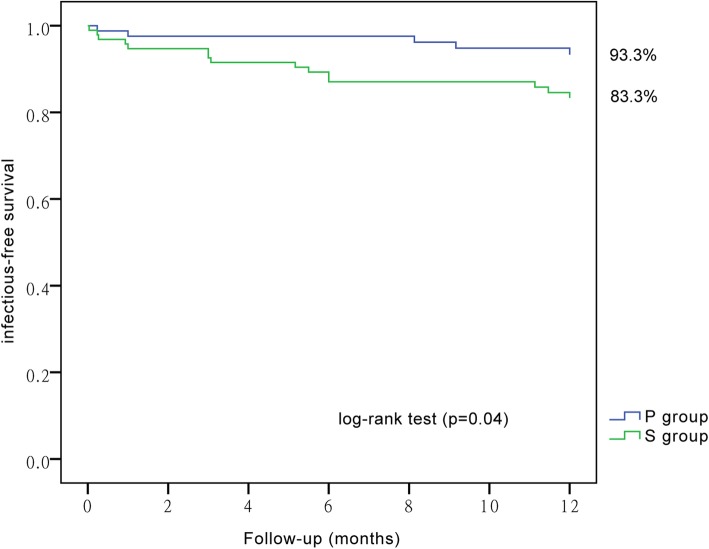
One-year infection-free catheter survival was better in patients undertaking percutaneous insertion. Patients were divided into two groups by insertion technique, i.e., percutaneous group (group P) and surgical group (group S). Follow-up period was one year. Then infection-free catheter survival was plotted by Kaplan–Meier curves. Log-rank test was performed to examine the significance

The one-year dysfunction-free catheter survivals were similar between the two groups, 71.08% (59 cases) and 74.74% (71 cases) for group P and S, respectively, *P* = 0.58. The one-year infection-free catheter survivals were also similar between the two groups, 75.90% (63 cases) and 68.42% (65 cases) for group P and S, respectively, *P* = 0.27. Similarly, the one-year dysfunction-and-infection-free catheter survivals showed no significance between the two groups, 65.06% (54 cases) and 63.16% (60 cases) for group P and S respectively, *P* = 0.79. The overall catheter survival was also similar between the two groups, 81.93% (68 cases) and 81.05% (77 cases) for group P and S, respectively, *P* = 0.88. The one-year infection-free catheter survival demonstrated the largest gap between the two groups (group P 75.90% vs. group S 68.42%), though not of statistical significance. However, a significant difference appeared in Kaplan-Meier analysis for one-year infection-free catheter survival between group P and S (by log-rank test *P* = 0.04, Fig. 2).

**Fig. 3:**
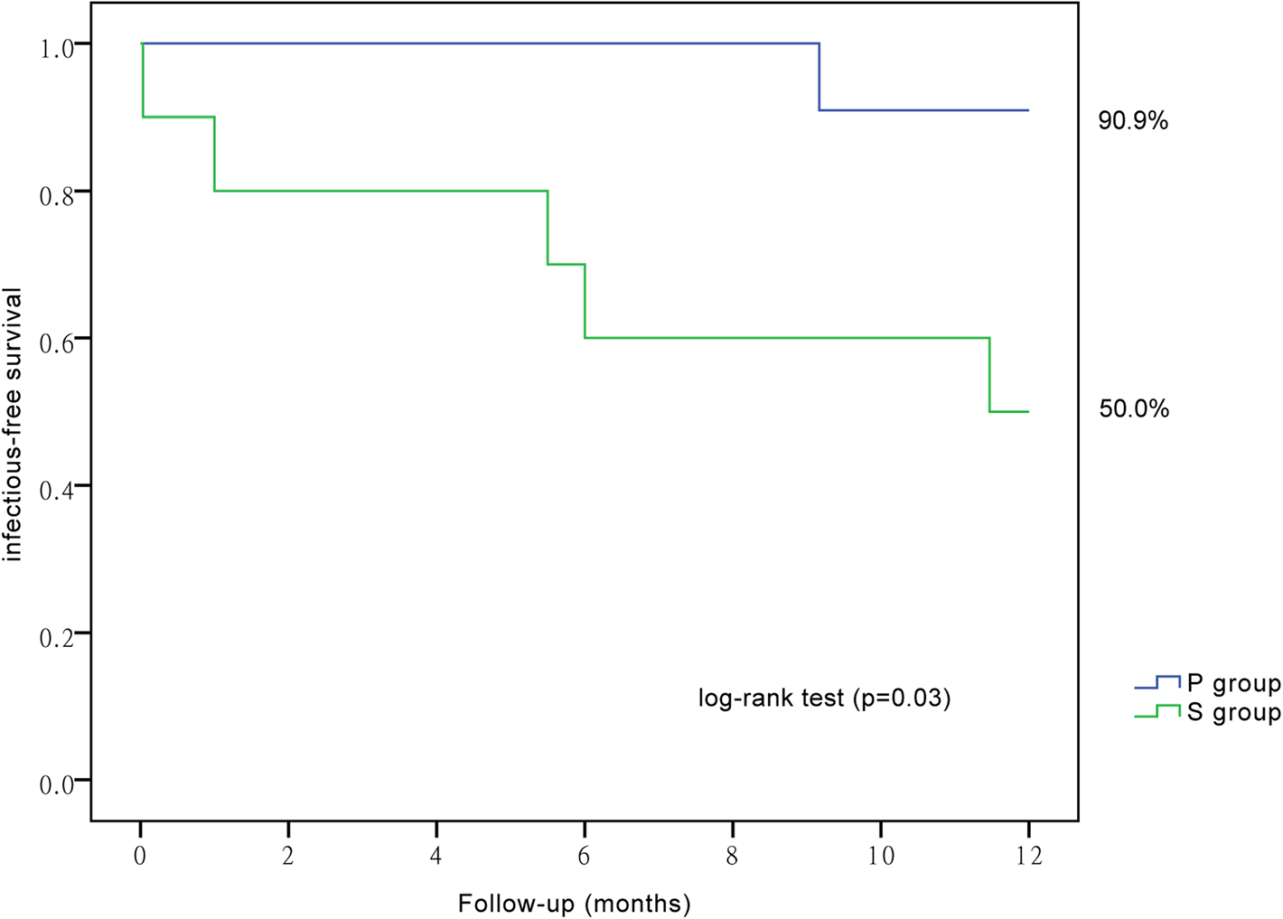
Among patients with a larger BMI, percutaneous technique presented a better one-year infection-free catheter survival than surgical technique. Patients were grouped by insertion technique, i.e., percutaneous group (group P) and surgical group (group S). Then subgroups were created according to patients’ BMI. This figure shows only patients in subgroup A (BMI > 28, 12 percutaneous and 10 surgical). Follow-up period was one year. Then Kaplan–Meier analysis with log-rank test was performed for infection-free catheter survival. BMI, body mass index

The results of subgroup analysis are shown in Table 4. Between different subgroups, the one-year infection-free catheter survival distribution was significantly different. Further, we discovered that in subgroup A the survival of group P (40.91%) was higher than that of group S (22.73%). The one-year dysfunction-free catheter survival, one-year dysfunction-and-infection-free catheter survival, and overall catheter survival distributions were similar between all subgroups. Kaplan-Meier survival analysis confirmed that the one-year infection-free catheter survival of group P was significantly higher than that of group S in subgroup A (by log-rank test *P* = 0.03, Fig. 3).

**Table 4:** Subgroup analysis for one-year catheter survival by BMI (Percutaneous versus Surgical)

one-year catheter survival	Percutaneous (*n* = 83)		Surgical (*n* = 95)		OR_MH_ (95% CI)	*P*-value
	BMI > 28 (*n* = 12)	BMI ≤ 28 (*n* = 71)	BMI > 28 (*n* = 10)	BMI ≤ 28 (*n* = 85)		
Dysfunction-free [n (%)]	9 (40.91)	50 (32.05)	9 (40.91)	62 (39.74)	0.99 (0.40 to 2.45)	0.98
Infection-free [n (%)]	9 (40.91)	54 (34.62)	5 (22.73)	60 (38.46)	3.04 (1.04 to 8.87)	0.03
Dysfunction-and-infection-free [n (%)]	8 (36.36)	46 (29.49)	5 (22.73)	55 (35.26)	1.52 (0.73 to 3.17)	0.26
Overall catheter survival [n (%)]	10 (45.45)	58 (37.18)	9 (40.91)	68 (43.59)	1.71 (0.41 to 7.13)	0.46

Variables are presented as number and percentage. The percentage is the proportion of survival cases in the relevant subgroup. Subgroup analysis was performed by stratified chi-square test. Catheter dysfunction-free survival was defined as total length of time from catheter insertion to revisionary surgery or removal due to mechanical dysfunction only. Catheter infection-free survival was defined as total length of time from catheter insertion to peritonitis or catheter related infections. Catheter dysfunction-and-infection-free survival was defined as total length of time from catheter insertion either to revisionary surgery or removal due to mechanical dysfunction, or to infectious complications (peritonitis, or catheter related infections). The overall catheter survival was defined as total length of time from catheter insertion to removal. Subgroup A: data in the two columns of BMI > 28, subgroup B: data in the two columns of BMI ≤ 28. *BMI* Body mass index

## Discussion

In the present study, total infectious complications of percutaneous insertion were significantly fewer than those of surgical insertion. Kaplan-Meier analysis demonstrated a significant higher one-year infection-free catheter survival in group P (93.3%) than in group S (83.3%). It should also be noted that despite the fact that group P was older with more co-morbidities, they had a lower rate of infectious complication and longer catheter survival.

In recent years, peritoneal dialysis has been widely used because of mortality benefits, improved quality of life, preserved residual renal function, lower cost, minimal infrastructure required and hemodynamic stability. PD may be a good option under certain circumstances such as hemodynamic instability, limited vascular access, active bleeding or bleeding tendencies. PD can also be used for acute kidney injury, heart failure, elderly patients, and urgent-start dialysis [7, 22–30]. As stated previously one of the key steps to having a successful PD program is having access to timely PD catheter placement [31]. Currently, there are several techniques available for PD catheter placement which include surgical, laparoscopic and percutaneous. Compared with other methods, percutaneous insertion is a simple procedure, with the benefits of quick recovery, earlier ambulation, and less delay in catheter placement [16, 32, 33]. In addition, because of avoiding a large peritoneal incision and the need for suturing, the percutaneous insertion can save much more time during the procedure [34, 35]. Some studies have reported that the mechanical complication rate of percutaneous insertion was similar to that of surgical and laparoscopic [10, 15, 33, 36], the infectious complication rate was lower [10, 36, 37], and survival was better [33]. Similar conclusions have been drawn in this study. The major concern with the percutaneous placement is that it is a “blind” technique with the risk of inadvertent puncture of the abdominal viscera and possible bleeding. However, the very low frequency of perforation reported in previous studies (0–1.3%) argued against the magnitude of this complication [38–42]. In the present study, none of the patients experienced a visceral injury or severe bleeding, and fewer patients in the percutaneous group had infectious complications. One of the most important points of this study was the fact that all of the percutaneous insertions were completed by nephrologists, which can reduce unnecessary procedures such as temporary hemodialysis catheters, and improve PD utilization by avoiding delays in catheter placement due to limited operating room slates and losing appropriate PD patients to hemodialysis [9, 11, 33, 43]. In addition, it has been shown that nephrologists taking ownership of catheter placement improves the success and growth of peritoneal dialysis programs [44].

Although there are many advantages using the percutaneous technique, most studies describe a BMI > 28 as a relative contraindication [12–16]. McDonald et al. [45] believed that obesity was a negative influencing factor of PD technique survival. Recently, there have been several studies which have described successful treatment of obese patients using peritoneal dialysis [46–49]. However, there is minimal information on PD catheter placement in this population. Singh et al. [50] evaluated a cohort of 315 patients and found that BMI was not an influencing factor of PD catheter survival, but all catheter studied were inserted by surgical placement. Krezalek et al. [51] conducted a cohort study using BMI as influencing factor, and found that obesity did not increase complications or shorten dysfunction-free catheter survival but only laparoscopic and open surgical catheter placement were included. Shanmugalingam et al. [52] utilized ultrasound assessment for selection of patients for percutaneous insertion of PD catheters, but obese patients were excluded. In fact, there was little knowledge about the outcomes of percutaneous insertion and overweight patients until now. The results of our study demonstrated that the outcomes of patients with a large BMI are similar to or better than their counterparts. This offers a possibility for the application of percutaneous catheter insertion in patients with BMI > 28.

Due to the short time of observation, some late complications might not be fully manifested, so the long-term outcomes such as death, were not included in the analysis. Kaplan-Meier analysis might overestimate the event rate due to the presence of competing risks, and yield biased results. This is a retrospective study, the choice of catheter insertion method was not randomized. The patients in group P were older and with more co-morbidities, but they had a lower rate of infectious complication and longer catheter survival. So this is a better explanation of the advantages of group P. However, a randomly designed, relatively large prospective cohort would be required to directly confirm the results obtained in the present study.

## Conclusions

Percutaneous insertion of peritoneal dialysis catheters appears to be a safe and effective technique for catheter placement, and it may be a good choice to the PD catheter placement for patients with a large BMI. However, a larger series of studies would be needed to verify the safety and efficacy of the technique.

## Data Availability

The datasets analysed during the current study are available from the corresponding author on reasonable request.

## Abbreviations

BMI: Body mass index
PD: Peritoneal dialysis
